# Science during lockdown – from virtual seminars to sustainable online communities

**DOI:** 10.1242/jcs.249607

**Published:** 2020-08-14

**Authors:** Francesca Bottanelli, Bruno Cadot, Felix Campelo, Scott Curran, Patricia M. Davidson, Gautam Dey, Ishier Raote, Anne Straube, Matthew P. Swaffer

**Affiliations:** 1 Institute of Chemistry and Biochemistry, Department of Biology, Chemistry and Pharmacy, Freie Universität Berlin, Thielallee 63, Berlin 14195, Germany; 2 Institut de Myologie, INSERM UMR974, Sorbonne Université, Paris 75013, France; 3 ICFO-Institut de Ciencies Fotoniques, The Barcelona Institute of Science and Technology, Barcelona 08860, Spain; 4 The Francis Crick Institute, 1 Midland Rd, London NW1 1AT, UK; 5 MRC Lab for Molecular Cell Biology, UCL, Gower Street, London WC1E 6BT, UK; 6 Centre for Genonmic Regulation (CRG), The Barcelona Institute of Science and Technology, Dr Aiguader 88, Barcelona 08003, Spain; 7 Universitat Pompeu Fabra (UPF), Barcelona 08003, Spain; 8 Centre for Mechanochemical Cell Biology & Warwick Medical School, University of Warwick, Coventry CV4 7AL, UK; 9 Department of Biology, Stanford University, Stanford CA 94305, USA

## Abstract

The COVID-19 pandemic has disrupted traditional modes of scientific communication. In-person conferences and seminars have been cancelled and most scientists around the world have been confined to their homes. Although challenging, this situation has presented an opportunity to adopt new ways to communicate science and build scientific relationships within a digital environment, thereby reducing the environmental impact and increasing the inclusivity of scientific events. As a group of researchers who have recently created online seminar series for our respective research communities, we have come together to share our experiences and insights. Only a few weeks into this process, and often learning ‘on the job’, we have collectively encountered different problems and solutions. Here, we share our advice on formats and tools, security concerns, spreading the word to your community and creating a diverse, inclusive and collegial space online. We hope our experience will help others launch their own online initiatives, helping to shape the future of scientific communication as we move past the current crisis.

## Abstract (precis)

**Summary:** A practical guide to organising sustainable and inclusive virtual seminar series.

## Introduction

The global wave of lockdowns triggered by the COVID-19 pandemic has resulted in a rapid proliferation of online research seminars (https://researchseminars.org/, https://thenode.biologists.com/list-of-virtual-talks-seminars-forums/events/, https://www.worldwideneuro.com/) as scientists around the world searched for ways to sustain a degree of scientific exchange during these unprecedented times. These virtual seminar series, varying widely in theme, organisational style and frequency, have been met with widespread success – it is becoming apparent that they will have a lasting impact on science communication beyond the current lockdowns (Bozelos and Vogels, 2020, eLife labs; Mill, 2020, FEBS network; Viglione, 2020a).

The appeal is clear (Abbott, 2020; Hampton et al., 2017; Welch et al., 2010). On the one hand, virtual science events can operate at negligible environmental and financial cost compared to traditional meetings (Achakulvisut et al., 2020; Sarabipour et al., 2020 preprint). On the other, organisers have the capacity to dramatically increase the number of opportunities for researchers, especially those at early stages of their careers, to present their work and attend top-quality seminars. In turn, these efforts can strengthen inclusive scientific networks for early career researchers, members of under-developed scientific communities and those otherwise unable to travel to international conferences (Achakulvisut et al., 2020; Sarabipour et al., 2020 preprint).

Many have long called for dramatic reforms to the structure and frequency of academic conferences and meetings, citing their increasingly untenable ecological footprint and the persistent inequality between scientific communities and access to scientific networks in different countries (Kier-Byfield, 2020, The Guardian Education; Spinellis and Louridas, 2013; Waruru, 2018, Nature news; Wilson and Biggs, 2016). With an unprecedented number of scientists attending (or delivering) their first virtual lecture this spring and summer, we are faced with an exciting opportunity to cement, within the academic landscape, these new ways to communicate science broadly and openly (Bidmon et al., 2020, Nature Career column; Haage, 2020; Viglione, 2020a; Weissgerber et al., 2020).

We present here a set of practical suggestions for anyone aiming to create an online seminar series for focussed research communities, drawing from our own diverse experiences in organising talks in the spring of 2020 for those investigating membrane trafficking (https://felixcampelo.wixsite.com/membranetrafficking), fission yeast research (https://researchseminars.org/seminar/pombeTalks), the cell biology of the nucleus (https://twitter.com/NucleusSciTalks), and molecular motors (http://mechanochemistry.org/whatson/MiQ/). We further discuss the directions we hope the scientific community at large will take to ensure the long-term sustainability of this broadly accessible format.

## Develop a theme around a recognisable identity

The theme of your new series needs to reflect at least a subset of the current interests of your community or field(s). You could aim to strengthen existing links, substituting for pre-existing in-person meetings, or instead leverage this moment to foster interactions between people of different fields, training and expertise that might not have shared a platform in the past. Creating a space that stimulates new discussions and collaborations can be particularly rewarding. Try to keep the theme coherent – too broad or too vague and you may not attract enough interest, but equally, too narrow and you will struggle to grow your community beyond a small base. If you focus your theme around an existing research community, you will already have a tailor-made audience. Ultimately, the very same audience will decide if your series is a success, so make sure to survey their interest! You will need to spread the word to grow your community – positive branding and use of social media will get you a long way. Box 1 contains some detailed recommendations in this regard.

**Box 1. Building an identity and brand**

**Survey your community.** Talk to colleagues, gauge interest through initial surveys shared on social media as well as through national or international scientific societies, and existing platforms for meetings, and use community mailing lists or Slack workspaces to confirm interest in your topic.

**Synergise with other organisers.** When choosing a theme, ensure you are not in competition with an existing seminar series. Bear in mind that you can propose joint meetings if an upcoming speaker might be of interest to multiple communities.

**Think about your brand.** This will help potential participants to recognize you and spread the word. You need a name, perhaps something catchy? Keep it short; it should be easy to remember and clearly convey the scope of your seminars. You could try to come up with a logo – be creative and have fun!

**Start developing your content.** Consider the type of speaker your audience would be interested in, then reach out to see if they would like to present. Remember that this is an opportunity to amplify the voices of scientists traditionally excluded from speaking at large conferences (because of funding issues, visa restrictions or other constraints). Also keep in mind that it is easier to generate interest if you already have speakers lined up. Circulate all the relevant information: the date and time, the format, sign up information and the first speakers. Include your logo, or an image relevant to your theme. Once you have generated interest with your first few speakers, you will need to keep the ball rolling and select more speakers. You may choose to personally invite each speaker or select from a pool of volunteers. In this case, make sure you include a call for speakers, and decide on a selection rubric (title or abstract).

**Get the word out.** Sign up for a seminar-specific email address, Twitter account, Slack account, website or any other resources you decide to use to announce your series. Advertise regularly to remind people of the talks and to foster interest. Post your speaker schedule early so people can plan to attend talks they are interested in. Write a few words about each speaker and share images related to their talk. Bear in mind that some platforms are not available in all countries, so make sure to diversify.

**Raise some funds.** Consider contacting a scientific society, a journal in your field, or companies with related expertise to see whether they would be interested in sponsoring the event. This is especially relevant if you require funding to subscribe to a premium version of an online tool or platform. Do not forget to ask your institution, it may already have a subscription ready for you.

## Find the right team, format and tools

Once set on your theme, you need three main ingredients to launch your initiative: your scientific audience and presenters, a committed team of organisers and the right online tools. You need peers willing to present their research, engage in discussions, provide feedback, or simply listen and learn.

You will need to find a set of like-minded volunteers to help you put it all together. Going it alone will almost certainly be more work and stress than you'd expect, and it is inevitable that at some point, you will need someone to cover responsibilities when other demands require your time and attention. This also provides a critical opportunity to reach out to researchers in other countries outside your immediate network who might provide complementary perspectives on scientific content and organisation.

Once you have formed a team, you have to choose a format that suits the needs and interests of your community (Table S1). In general, people are unlikely to commit more than 1 h during a working day to a webinar. Decide whether you want long keynote-style seminars (for example, 45 min talk plus 15 min discussion) or multiple shorter talks (for example, 2×15 min plus 15 min discussion). No single format suits all purposes, and you don't have to stick to the same format throughout your series. So, how do you start? One natural choice would be to run a simple periodic webinar series. How often should the talks be? If you foresee difficulties in finding speakers, schedule seminars every fortnight or once a month. Do you expect many to volunteer or agree to speak if invited? In this case, try a weekly format. Be flexible: most of us expect a decrease in frequency as lockdowns ease, and some seminar series have already adapted their format and frequency (for example, the *Nucleus Science Talks* actually increased frequency from once to twice a week, and now periodically alternates between one long talk and two shorter talks per session).

The next step is more technical, but equally important: finding the right online tools to make it all happen – to organize, announce and host the webinars (as well as their recordings, if possible, for asynchronous viewing) and the ensuing discussions. Maximizing your use of the available tools can make all the difference in your seminar series becoming a success. There is also a great opening here to foster a sense of online community outside of the live-streamed talks. If there are more questions than can be answered during the webinar, offer breakout rooms to continue discussions at the end. Versatile communication platforms, such as Slack, are perfect for hosting follow-up Q&A sessions and discussion in the hours and days after a seminar. Box 2 has useful information and tips about the currently available tools and how to make the most of them, as well as our perspective on best practices for keeping your seminar secure.

**Box 2. Getting the most out of online tools**

**Create a database.** Centralize registration using a form-building service (such as mailchimp, JotForm, Google Forms or Eventbrite) and a simple website or twitter account for announcements. Most options are entirely free or open source (or both).

**Host your online seminar.** Your choice of videoconference software should be multiplatform (Windows, macOS, iOS, Android, Linux), free for attendees, and able to handle a large number of participants. It should also allow for guest video/audio control by the hosts, for recording by only the host, and, ideally, be secured with end-to-end encryption. Options include Zoom, Skype, Microsoft Teams, Cisco Webex, Jitsi or Google Meet.

**Post your recorded seminar online.** Many seminars are posted on YouTube after the live seminar. YouTube allows videos to be made private so that they cannot be found using search engines. It also offers automatic closed captioning, which is not perfect, but can be manually corrected.

**Connect your community.** To boost interaction and discussion amongst the group, you can combine the videoconference tool with other platforms that enable fast and dynamic communication. Tools such as Slack (the free version has all the features you need) are very powerful, providing topic-based communication channels. We would advise you to first become familiar with the platform, create a full set of channels, and provide instructions to the members, before releasing it to the outside world (we provide suggestions in Box 3).

**Keep on top of security.** Openness in an online format is not risk-free. Unfortunately, the hijacking of online seminars (colloquially known as ‘zoom bombing’) has become increasingly common. This can be avoided by using webinar formats in which audience participation requires explicit host permission. We recommend password control and advise against posting login details directly to social media or publicly archived mailing lists. If the worst does happen, some platforms will allow you to remove attendees. Become familiar with these functionalities in advance so you can act quickly if required.

**Experiment with new tools.** To include a networking aspect with the seminars you can try ‘break-out rooms’ – several tools support them. Tools such as Slido and Miro can help you collect real-time data or work collaboratively, although free versions only provide limited functionality – be sure to consider data privacy and security issues and platform stability.

## Make the space inclusive, equitable and safe

By default, online science events remove known barriers to conference participation, such as limited travel funds, difficulties to obtain visas or personal circumstances that limit travel (Sarabipour et al., 2020 preprint) (e.g. family commitments, disability). However, you still need to make your series widely accessible. Ideally, attendance to online seminars should remain free, to allow everyone who is interested to participate. The main costs incurred are licensing fees for webinar platforms and, if not available through your institution, a sponsor might be willing to associate with the series (for example, the *Motors in Quarantine* series has received funding from Journal of Cell Science for this purpose).

The absence of a fee does not automatically drive inclusivity, and we strongly recommend committing to a diversity statement outlining the core values of inclusivity of your seminar series. This statement should explicitly recognize that diversity is a source of strength, and detail the steps that the seminar organizers are taking, and will continue to act upon in the future, to increase and maintain diversity. We've provided examples that we use in our seminar series (Box 3). As with any well-designed scientific event, you should provide opportunities for early career researchers, such as PhD students and postdocs, and commit to supporting a gender balance (Langin, 2019, Science Careers). Detailed guidance on achieving gender balance has been covered previously (Else, 2019; Martin, 2014; Vallence et al., 2019).

**Box 3. Additional resources**

**Sample Slack guidelines.** If using a communication platform like Slack, it is best to create and organise channels, and guidelines for posting behaviour, up front rather than being forced to reverse-engineer them down the line. https://osf.io/vz34p/wiki/Sample%20Slack%20guidelines/.

**Sample diversity statement.** Your diversity statement should clearly identify the goals of the community and specific actions taken to foster and support diversity and inclusivity; provide a mechanism for feedback. https://osf.io/vz34p/wiki/Sample%20diversity%20statement:%20pombeTalks%20and%20pombeSlack/

**Sample code of conduct.** Your code of conduct should identify best practice as well as mechanisms for seeking redressal in the case of harassment or inappropriate behaviour within the context of your forum. https://osf.io/vz34p/wiki/Sample%20code%20of%20conduct/. A permanent archive of these resources will be maintained at: https://osf.io/vz34p/ (doi: 10.17605/OSF.IO/VZ34P).

If the speakers are happy for their talks to be recorded, they can be made available online for all registered attendees, or even the public, to view at a convenient time of their choosing. This is especially important for participants who cannot attend live seminars due to other commitments, time zone differences or insufficient internet bandwidth. However, you need to make sure that speakers are comfortable with how you share the recordings and allow them to decide for how long the video will be posted, or to opt out of posting the video entirely. Posting the video for a few days is often a good compromise for those speakers who are not comfortable with long-term hosting. Consider providing subtitles (platforms such as YouTube provide automatic closed captioning, which can be manually edited if necessary). Subtitles are not only helpful for people with hearing loss, but also those whose first language is not English, or those who experience background noise or cannot turn on the sound on their device.

If your online seminars are not recorded and only available for live viewing, it is particularly important to choose a time that allows attendance across the globe. Most online series are scheduled between 15:00 and 17:00 GMT as a best compromise to maximise attendance from the west coast of the Americas to East Asia and Oceania. In informal surveys conducted within our communities, we have found that the vast majority of participants (>80%) prefer to attend live seminars when possible. Therefore, if you do make recordings available, it is still important to schedule a live-viewing time that maximises real-time participation and thus promotes interactions during and immediately after the talk.

Often the most rewarding seminars are those presenting unpublished data, but speakers take the small risk that some in the audience may not treat their data as confidential. This risk is not exclusive to online seminars, but unauthorised screenshots or recordings are harder to regulate than photography in a conference hall. The best way to mitigate this risk is to have clear community guidelines from the outset and regularly remind attendees of their obligation to respect them (see Box 3 for an example of a Code of Conduct statement). Ultimately, if you can foster a strong, diverse and inclusive online community with collegial discussions, this will help to generate the goodwill to respect such rules.

## Assess and adapt

**Fig. 1.**
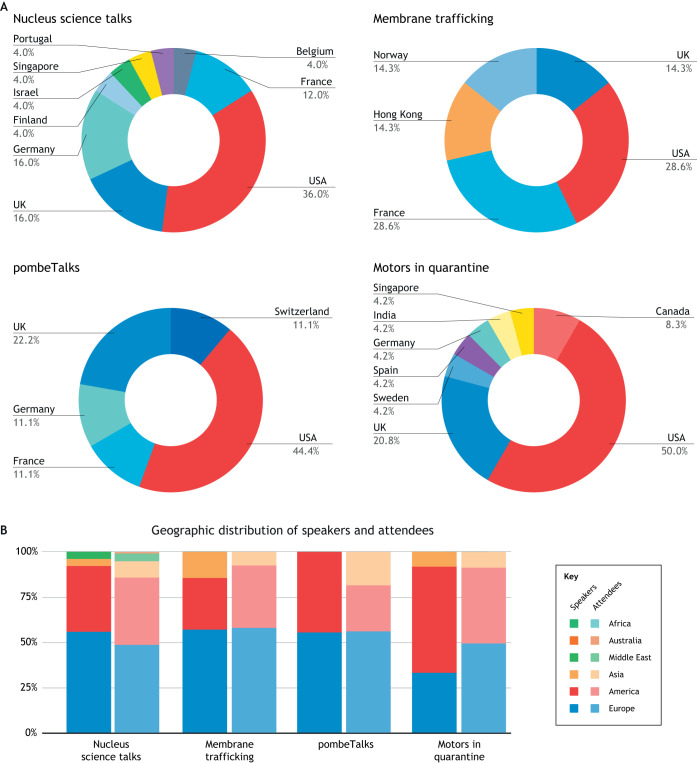
**Speaker and audience demographics.** (A) Geographic distribution of speakers across the four different online seminar series, until the 20th of June 2020. (B) Combined geographic distributions of speakers and attendees for the four different online seminar series, until the 20th of June 2020. Colours indicate continents. Underlying data provided in Table S1.

As you host and attend more online seminars, you have the opportunity to innovate in a data-informed manner. You can run surveys to decide on a sustainable frequency of online events, different seminar formats and whether these formats can co-exist in the same organisational space. Some platforms will allow you to gather geolocalization data on your participants (often at a cost). From which institutions and countries do most participants attend? This data can feed back into conference planning to improve participation and inclusivity in the future (Fig. 1; Table S1).

Although surveying your participants can help identify barriers to participation, you will probably have to make a committed effort to draw in under-represented communities: by definition, members of these communities might not form part of your social media and traditional networks. Contact local scientific societies and liaise with organisations that promote local and international scientific communication. In surveys of our participants, we found that we had few (and sometimes no) participants from South America, Africa and Asia (Fig. 1), with the largest proportion of participants located in Europe. Geolocalisation data obtained by the *Motors in Quarantine* seminar series indicates a mismatch between the physical locations of authors publishing papers on cytoskeletal motors and the locations of seminar attendees, with researchers from Asia and South America under-represented amongst the attendees (Royle, 2020, Quantixed Blog). The precise reasons for this mismatch, and for the over-representation of European attendees among our series in general, are currently unclear. However, we are now working towards better disseminating information about our seminar series in under-represented geographic areas by reaching out to local research communities and scientific organizations.

## Final thoughts on launching a series

Virtual seminars and online communities are powerful tools to keep scientists connected, share findings effectively and set up collaborations to advance research. This might sound like a mammoth task landing on the shoulders of those taking the first organisational steps. However, it is important to remember to have fun! If you are thinking about starting an online community or seminar series, you are likely already bursting with enthusiasm and passion for your research topic. Find like-minded colleagues, re-read this article for tips and get started. You don't have to be an independent group leader or well-established in the field. Most of us, in fact, are early-career researchers that wanted to nurture a sense of belonging to a community in these times of confinement. If you are not convinced yet, think about it as a chance to create your own personal dream conference. Reach out to authors of papers or pre-prints you found interesting and enjoy their talk from the comfort of your sofa. This is all done without the hassle of having to travel the other side of the world and with the bonus of a cup of coffee in your hands, finally solving that ‘no drinks in the auditorium’ problem!

Do not underestimate the time commitment required to create such a platform from scratch, however. You will have to organise seminars, design posters, manage the social media accounts and you will receive a barrage of requests for technical assistance. However, with the guidance provided here, we hope that we will mitigate for you many of the challenges we faced when we first set up our platforms. Good luck!

## The long view – online seminars can evolve to shape the future of online scientific exchange

Online science events are here to stay (Gichora et al., 2010; Viglione, 2020a,b). In a recent Nature reader poll, more than 80% of respondents said that they would be in favour of some scientific conferences remaining virtual after the COVID-19 pandemic (No author, 2020). With respect to our own seminar series, our online communities are keen to continue with seminars even after the current crisis, with participants emphasising the advantages over in-person events: the low cost and commitment, the availability of speakers on other continents, and the ease of participation.

One downside of the online format is the loss of face-to-face networking opportunities. To promote interactions between researchers at different stages of their careers in both formal and informal settings, virtual meeting rooms can replicate meet-the-speaker tables at conferences, or random allocation into ‘breakout’ rooms or scientific speed-dating sessions (Vaggi et al., 2014) for unplanned encounters that may lead to the unforeseen but productive dialogues we all enjoy.

In the longer term, we believe that the future of the format lies in moving from seminars to creating community-driven interaction spaces. Once a community is set up, why remain restricted to specific events at certain times of the year? Think of this as a space where your community will gather and connect, to pursue shared interests or activities, both serious and fun. These could include journal clubs, stand-alone mini-conferences, industry partners showcasing their wares, platforms to connect job seekers with potential employers, and virtual social events, to name just a few options.

In doing so, such online spaces will supplant many of the functions currently served by in-person conferences (Sarabipour et al., 2020 preprint). In fact, conferences themselves will of necessity adapt to this new reality, creating hybrid events with in-person talks streamed on online channels and online talks streamed in conference venues. The grassroots, bottom-up organisational principles that drive successful online seminar series will evolve into community spaces that properly represent the incredible diversity and global, interconnected nature of the scientific enterprise.

## Supplementary Material

Supplementary information
