# Supplementary material for: Science during lockdown – from virtual seminars to sustainable online communities
Source: J Cell Sci. 2020 Aug 14;133(15):jcs249607. doi: 10.1242/jcs.249607 (PMC7438008; doi:10.1242/jcs.249607)
Supplement: Supplementary information [file joces-133-249607-s1.pdf]

Table S1. Online seminar series in numbers.

Click here to Download Table S1

Journal of Cell Science • Supplementary information
